# Fascin in Gynecological Cancers: An Update of the Literature

**DOI:** 10.3390/cancers13225760

**Published:** 2021-11-17

**Authors:** Ishita Gupta, Semir Vranic, Hamda Al-Thawadi, Ala-Eddin Al Moustafa

**Affiliations:** 1 Department of Basic Medical Science, College of Medicine, QU Health, Qatar University, Doha 2713, Qatar; ishita.gupta@qu.edu.qa (I.G.); svranic@qu.edu.qa (S.V.); halthawadi@qu.edu.qa (H.A.-T.); 2 Biomedical and Pharmaceutical Research Unit, QU Health, Qatar University, Doha 2713, Qatar; 3 Biomedical Research Centre, QU Health, Qatar University, Doha 2713, Qatar

**Keywords:** fascin, gynecological cancer, ovarian cancer, cervical cancer, biomarker

## Abstract

**Simple Summary:**

Fascin, an actin-binding protein, is upregulated in different types of human cancers. It is reportedly responsible for increasing the invasive and metastatic ability of cancer cells by reducing cell–cell adhesions. This review provides a brief overview of fascin and its interactions with other genes and oncoviruses to induce the onset and progression of cancer.

**Abstract:**

Fascin is an actin-binding protein that is encoded by the *FSCN1* gene (located on chromosome 7). It triggers membrane projections and stimulates cell motility in cancer cells. Fascin overexpression has been described in different types of human cancers in which its expression correlated with tumor growth, migration, invasion, and metastasis. Moreover, overexpression of fascin was found in oncovirus-infected cells, such as human papillomaviruses (HPVs) and Epstein-Barr virus (EBV), disrupting the cell–cell adhesion and enhancing cancer progression. Based on these findings, several studies reported fascin as a potential biomarker and a therapeutic target in various cancers. This review provides a brief overview of the FSCN1 role in various cancers with emphasis on gynecological malignancies. We also discuss fascin interactions with other genes and oncoviruses through which it might induce cancer development and progression.

## 1. Introduction

Fascin, also known as *fascin-1* (*FSCN1*) or actin-bundling protein-1, is a globular filamentous actin-binding protein belonging to the actin cytoskeletal protein family [1]. Molecular cloning techniques showed fascin to be highly conserved during the course of evolution. Furthermore, fascin is homologous to several species, including the Drosophila singed gene [2], Xenopus [3], and mouse [4]. *FSCN1* plays a crucial role in the assembly and maintenance of various cellular structures, including filopodia, stress fibers, lamellipodia, invadopodia, dendrites, and spiky protrusions underlying the plasma membrane [5,6,7,8]. In addition to its role in maintaining actin structure, *FSCN1* regulates several cellular physiological processes, including cell-to-cell interactions, cell-to-matrix adhesion, cell motility, cell migration and invasion as well as focal adhesion dynamics, histone methylation, and gene transcription [9,10,11,12,13].

There are three isoforms of *FSCN* (*FSCN1*, *FSCN2*, and *FSCN3*) present in humans as well as other vertebrates. Of the three isoforms, *FSCN1* is expressed during development in the mesenchymal and nervous tissues and is the most extensively studied form of fascin [14]. On the other hand, *FSCN2* is expressed in hair and retinal cells [15,16], while *FSCN3* is present in the testis and developing spermatozoa [17]. Both *FSCN-2* and *-3* are homologous to *FSCN-1* by 56% and 29%, respectively.

While in normal tissues there is low or lack of *FSCN1* expression, in transformed epithelial cells and carcinomas, it is highly expressed [18]. In different cancers, including colon, pancreatic, breast, lung, esophagus, stomach, skin, and ovarian cancers, elevated expression of *FSCN1* is associated with increased metastatic potential and poor prognosis, indicating its role as a candidate potential biomarker and therapeutic target [19]. Here we discuss the current knowledge of *FSCN1* and its underlying mechanisms in order to elucidate its multiple roles in the onset and progression of gynecological cancer.

## 2. Structure of Fascin

In humans, the *FSCN1* (Gene ID: 6624) encodes a distinct 493 amino acid polypeptide and is located on the short arm of chromosome 7, encompassing ~14 kb of DNA including 5 exons. 

X-ray crystal structure and sequence analyses revealed *FSCN1* to consist of four tandem β-trefoil domains (residues 8–139, 140–260, 261–381, and 382–493) organized as two twisted lobes (β-trefoil 1 and 2; β-trefoil 3 and 4), each of which includes six 2-stranded β-hairpins [20]. Studies demonstrated that all the four β-trefoil domains of *FSCN1* play a role in actin binding; while the actin-binding site (ABS)-1 involves the β-trefoil 1 and 4, ABS-2 includes residues from β-trefoil 1 and 2, and ABS-3 involves β-trefoil 3 [21]. Microtubules interact with *FSCN1* directly via β-trefoil 2, plausibly inhibiting ABS-1 [12]. The ABS-1 region is located in the first beta-trefoil domain between amino acids 33–47 and consists of a highly conserved site (Ser39) in the center of ABS-1, which can be phosphorylated by protein kinase C (PKC); studies have indicated Ser39 phosphorylation inhibits *FSCN1* activity [22,23]. In addition to Ser39, *FSCN1* consists of another phosphorylation site, Ser274; mutation of Ser274 to alanine is also involved in inhibiting fascin-bundling activity; however, Ser274 does not lie in the region of ABS1-3 [21,24,25]. On the other hand, ABS-2 consists of two lysine residues (K247/K250) located at the lysine-rich loop at the β-trefoil domain 2 of *FSCN1* (between amino acids 241–250) [26]; monoubiquitylation of *FSCN1* at K247/K250 stimulates bundle assembly [27]. The postulated location for the other ABS is between amino acids 277–493 [10]. 

## 3. Functions of Fascin

Physiologically, *FSCN1* is expressed at comparatively low levels in normal cells as compared to malignant cells. Therefore, the distinct roles of *FSCN1* in both cell types are important to review. 

### 3.1. Function of Fascin in Normal Cells

As mentioned in the section above, *FSCN1* is an actin-bundling protein and crosslinks actin filaments via the three binding sites [5,7,21]. *FSCN1* plays a role in the formation and stabilization of several cellular protrusions (microspikes, lamellipodia, and filopodia) [5,7,21] which are essential for cell-to-cell adhesion, cellular interaction, motility, and migration [9,10,11]. Additionally, *FSCN1* also regulates focal adhesion dynamics in multiples types of cells and is partially dependent on the canonical actin-bundling function of *FSCN1* [6,12]. Moreover, during normal development, *FSCN1* controls cellular processes including cell migration, neurite cone extension, and dendrite formation [19,28,29]. During dendritic cell maturation, *FSCN1* is highly expressed; *FSCN1*-dendritic cells aid in effectual interaction and present antigens to T cells, thus playing a vital role in adaptive and innate immunity [30].

On the other hand, *FSCN1* binds to microtubule cytoskeleton and regulates focal adhesion dynamics and cell migration [12]. Interruption in *FSCN1* and microtubule interaction leads to stability of cell adhesion and reduces cell migration [12]. During cell migration and invasion, *FSCN1* interacts with nesprin-2, a nuclear envelope protein to promote nuclear deformation and mobility [31]. Nevertheless, the phosphorylated form of *FSCN1* is present in the nucleus and controls histone methylation and gene transcription by interaction with H3K4me3, the H3K4 methyltransferase core subunit RbBP5 form [13]. Nonetheless, *FSCN1* regulates extracellular vesicle release [32]. 

*FSCN1* is expressed during mouse embryonic development; however, its expression patterns are widely conserved in human tissues [14,28]. In Drosophila oogenesis, *FSCN1* also plays an important role in delamination during border cell migration by modifying the localization of E-cadherin in the border cells [33]. During embryogenesis, as compared to adults, *FSCN1* is significantly expressed during development. In adults, *FSCN1* is absent or at low levels in normal epithelial cells, and its expression is limited to the neuronal, endothelial, mesenchymal, dendritic, and immune cells [18]. During embryogenesis, *FSCN1* is largely expressed in the nervous systems (neuroblasts, melanoblasts, mesenchymal tissue, microcapillary endothelial cells, and antigen-presenting dendritic cells) [18,34,35]. 

### 3.2. Function of Fascin in Cancer Cells

When epithelial cells undergo the transformation, *FSCN1* expression levels are highly elevated [36]. *FSCN1* overexpression is documented in the majority of cancers, including ovarian [37], breast [38], colon [39], pancreatic [40,41], glioma [42], melanoma [43], leukemia [44], lymphoma [45], and esophageal squamous cell carcinoma [46]; however, the underlying molecular mechanisms of *FSCN1* activation during the onset and progression of cancer are still understated. Although mutations or amplification of *FSCN1* gene are not common, hypomethylation of *FSCN1* promoter has been found in normal epithelium and cancer cells alike [46], indicating the lack of epigenetic aberration of *FSCN1* in cancer.

The role of *FSCN1* in cancer was first described in breast cancer by Grothey et al. [47], where the authors showed that overexpression of *FSCN1* induced aggressive phenotype. Several additional investigations also reported the role of *FSCN1* in other types of cancer and its association with an aggressive phenotype, poor prognosis, and short survival [19,36,48,49]. An earlier study found significant overexpression of FSCN1 in human epithelial tumors (lung, cervical, ovarian, esophageal, pancreatic, gastric, hepatocellular, colorectal, breast, nasopharyngeal, and laryngeal carcinomas) in comparison with their corresponding normal tissues, leading to the conclusion that overexpression of FSCN1 correlates with tumor occurrence and progression [50]. In this regard, it was found that *FSCN1* can promote cancer progression through both canonical and non-canonical pathways by triggering cancer proliferation, migration, invasion, and metastasis [19,36].

There are several functions by which *FSCN1* promotes cancer progression via inducing cancer cell growth, proliferation, migration, invasion, and metastasis [19,36]. In vitro findings reported contrasting roles of *FSCN1* in cell growth and proliferation. While upregulated expression of *FSCN1* was found to be correlated with enhanced cell proliferation in different cancer cell lines [51,52,53], few studies reported no significant cell proliferation in *FSCN1*-transduced cancer cells [54,55]. While one study in the aggressive breast cancer cell line, MDA-MB-231, reported stimulated *FSCN1* expression to provoke cell proliferation [51], other investigations by Al-Alwan et al. [56] and Heinz et al. [57] did not report a significant effect of transduced *FSCN1* expression on MDA-MB-231 cell proliferation. Similarly, in the non-small lung cancer cell line, A549, it was reported that enhanced *FSCN1* has a light impact on cell proliferation [58]; however, another study using A549 cells reported that *FSCN1* could enhance cell proliferation via the YAP/TEAD signaling pathway [52]. Moreover, knockdown of *FSCN1* leads to the inhibition of the A549 cell growth and proliferation via the MAPK signaling pathway [59].

On the other hand, one of the principal functions of *FSCN1* contributions to cancer progression is the actin-bundling function [6]. Alterations in the dynamics of microtubules, including their expression and stability, have been shown to lead to cancer invasion and metastasis [57,60]. *FSCN1* also facilitates mechano-transduction by interaction with the LINC complex, which is responsible for cell invasion and migration [31,61,62]. The other function includes the role of *FSCN1* within the nucleus to control nucleolar size and morphology [63], in addition to nuclear actin [64,65], chromatin regulation, and assembly [13]. Thus, deregulation in these functions contributes to cancer development and progression [65,66,67]. Additionally, *FSCN1* also regulates oxidative phosphorylation of the mitochondria and metabolic stress resistance, thus inducing cancer metastasis [54].

Moreover, in vitro data also demonstrated the role of *FSCN1* in inducing cancer cell migration by promoting filopodia formation and epithelial-mesenchymal transition (EMT) [68] in various cancer cells, including ovarian [37], oral [53], hypopharyngeal [69], osteosarcoma [70], and pancreatic cancer cells [41]. *FSCN1* induces filopodia and invadopodia stability and formation [7]. In addition, it enhances the expression of matrix metalloproteinases (MMPs) [41,69], thus promoting cancer cell motility and invasion. On the other hand, in vivo studies using different models also reported *FSCN1*-induced migration and invasion. In mouse and zebrafish models, *FSCN1* expression was found to induce colon tumor invasion [71,72]. Similarly, breast cancer in vivo models reported elevated *FSCN1* expression to induce cancer cell metastasis to the lung [40]. In nude mice models, overexpression of *FSCN1* was linked with metastasis in pancreatic [73], renal [74], and colorectal carcinomas [75]. Additionally, in severe combined immunodeficiency (SCID) mice, enhanced *FSCN1* expression provoked lung metastasis in osteosarcoma cells [70]. Furthermore, previous studies reported that *FSCN1* could promote cancer progression by inducing chemoresistance in cancer cells as well as controlling metabolism and contributing to a de-differentiated and more stem-like state [76]. 

### 3.3. Mechanisms of Fascin Deregulation 

**Figure 1:**
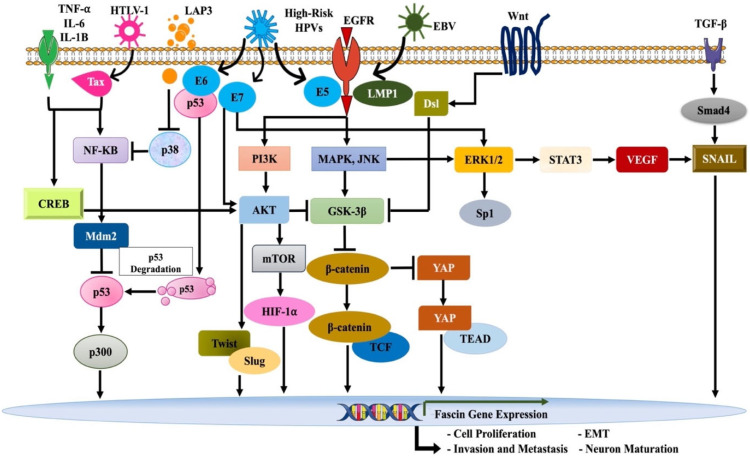
Mechanisms of *FSCN1* deregulation. Several factors, including EGFR, TGF-β, and interleukins, in addition to oncoviruses, trigger key pathways including CREB, ERK1/2, JNK, STAT3, PI3K, MAPK, and NF-κB to deregulate *FSCN1* expression and stimulate underlying mechanisms for cancer progression.

Numerous transcriptional factors (TFs) interact and bind to the *FSCN1* promoter regions, thus regulating *FSCN1* expression. It was reported that *FSCN1* transcriptional activity is controlled by the promoter region (−219/+114); in breast and colon cancer, CREB and aryl hydrocarbon receptors (AhRs) bind with the −219/+114 promoter region [77]. On the other hand, in human oral cancer, Lee et al. [78] reported that interleukin (IL)-1β triggered phosphorylation of several key players, including CREB, ERK1/2, JNK, and NF-κB, thereby inducing *FSCN1* expression and promoting invasion (Figure 1). Furthermore, cytokines (IL-6 or tumor necrosis factor-alpha (TNF-α) trigger the NF-κB and STAT3 pathways, which are essential for enhancing *FSCN1* expression in cancer [79,80,81] (Figure 1). In gastric cancer, one study reported that Fas signaling induces *FSCN1* expression via the STAT3 pathway [82]; another study demonstrated that galectin-3 regulates the GSK-3β/β-catenin/TCF-4 signaling pathway, thus triggering *FSCN1* expression [83] (Figure 1). In colorectal cancer, the β-catenin-TCF signaling pathway was reported to regulate *FSCN1* transcription [84]; however, the studies in breast and colon cancer failed to report the regulation of *FSCN1* expression via β-catenin-TCF signaling [77,85]. Another study in colorectal cancer demonstrated loss of p53 to induce *FSCN1* expression via the NF-κB pathway [86] (Figure 1). Moreover, in esophageal squamous cell carcinoma, overexpression of the epidermal growth factor (EGF) increased specificity of protein 1 (Sp1) phosphorylation and activated the ERK1/2 pathway, thus enhancing *FSCN1* expression [87] (Figure 1). Furthermore, the snail family of transcription factors was reported to be involved in *FSCN1* transcription; SNAIL2 expression induced *FSCN1* expression in human colon and pancreatic cells [40]. In head and neck cancer cells, SNAIL2 was found to directly bind to the *FSCN1* promoter, inducing *FSCN1* expression (Figure 1) [88]. On the other hand, in hypopharyngeal and pancreatic cancers, HIF-1α was reported to induce the overexpression of *FSCN1* [41,69] (Figure 1). Nevertheless, a study by Megrioni and colleagues [89] demonstrated the overexpression of *FSCN1* in NT2 cells, known to be deficient of the CREB-binding protein during neurogenesis, indicating a viral role of *FSCN1* in the formation of mature neurons (Figure 1).

Similar to transcription factors regulating *FSCN1* expression, microRNAs (miRNAs) are known to bind to the 3′ untranslated region (UTR) of *FSCN1* and regulate its expression in several human cancer tissues and cell lines, including breast, lung, liver, colon, cervix, prostate, pancreatic, hepatocellular, esophageal, and nasopharyngeal [90,91,92,93,94,95,96,97,98,99,100,101]. On the other hand, loss of miRNAs -133a and -145 was found to enhance *FSCN1* expression, thus stimulating cancer cell growth, proliferation, migration, and invasion along with the inhibition of apoptosis [90,91,92,93,94,96,97,98,99,100,101]. In addition, *FSCN1* is directly targeted by several miRNAs in different cancers. Thus, miRNA-24 targets *FSCN1* in nasopharyngeal and prostate cancer [95,102], *FSCN1* is targeted by miRNA-143 in esophageal [103] and miRNA-326 in lung and gastric cancers [104,105], respectively. Yu et al. demonstrated that the loss of miRNA-663 in colorectal cancer cells induced *FSCN1* expression [106]. Chen et al. reported overexpression of miRNA-451 to enhance *FSCN1* expression via the inhibition of AMPK and activation of mTOR signaling [107]. 

### 3.4. Role of Oncoviruses in Fascin Deregulation 

Previous studies have shown potential deregulation of *FSCN1* via viral oncogenesis (Figure 1). In this regard, overexpression of *FSCN1* was reported in Epstein-Barr virus (EBV)-induced lymphoblastoid cell lines [108]. More specifically, LMP1, one of the oncoproteins of the EBV, was found to stimulate *FSCN1* in lymphocytes via NF-κB signaling, contributing to lymphocyte migration and invasion [44]. While *FSCN1*-negative Hodgkin’s lymphoma-derived cell lines also show upregulated *FSCN1* levels [44], LMP1-negative Burkitt lymphoma-derived cell lines are negative for *FSCN1* expression [109]. Moreover, a study by Liu et al. in EBV-positive nasopharyngeal carcinoma reported that enhanced LMP1 levels and phosphorylated STAT3 elevated *FSCN1* expression and was associated with lymph node metastasis and higher proliferation index of the cancer cells [110]. Since LMP1 is present in epithelial cells and is known to have a potential role in EBV production [111], continuous expression of *FSCN1* can help in EBV release. Moreover, the transmission of EBV to epithelial cells is dependent on NF-κB signaling [112], which is one of the major factors for effective *FSCN1* induction. LMP1 of EBV can induce expression of *FSCN1* at both mRNA and protein levels in lymphocytes [113]. Studies by Mohr et al. [44,113] demonstrated that the inhibition of NF-κB signaling using a chemical inhibitor of IκB kinase β (IKKβ) or cotransfection of a dominant-negative inhibitor of IκBα (NFKBIA) decreased both *FSCN1* levels and the invasive ability of EBV-transformed lymphoblastoid cells. Moreover, the study showed that the knockdown of *FSCN1* by two different small hairpin RNAs reduced invasion of lymphocytes [113]. Similarly, in LMP1-positive Jurkat T lymphocytes, *FSCN1* is reported to induce cell migration [114]. In colorectal cancer, upregulated *FSCN1* expression was reported in LMP1-positive samples and was associated with moderately to poorly differentiated adenocarcinomas [115]. The study suggested that Wnt/β-catenin signaling regulates epithelial-mesenchymal transition (EMT) induced by *FSCN1* in LMP1-positive cancers to provoke cancer progression (Figure 1) [115]. Likewise, in EBV-associated gastric cancer, upregulated Smad4/FSCN1 expression significantly correlated with larger tumor size, higher histological grade, lymph node involvement, vascular invasion, and poor clinical outcome [116]. 

*FSCN1* upregulation was also correlated with other oncoviruses. Kress et al. [117] reported that Tax, the oncoprotein of leukemia-inducing retrovirus HTLV-1, could upregulate *FSCN1* expression through the regulation of the NF-κB signaling pathway (Figure 1). A recent study in adult T-cell leukemia/lymphoma (ATLL) with HTLV-1–infected Hodgkin and Reed-Sternberg–like cells found elevated *FSCN1* expression [118]. Gross and colleagues [119] further found *FSCN1* to play a vital role in the transport of viral proteins to budding sites and promote HTLV-1 transmission.

*FSCN1* was also reported to be associated with high-risk HPV to enhance cancer progression. It is well-known that high-risk HPVs are major players in the onset and progression of cervical cancer and correlate with lymph node and vascular invasion and the tumor size [120,121]. More specifically, Yasmeen et al. [122] investigated the expression of oncogenes, including *FSCN1*, in cervical cancer. In this study, the use of Src/Abl inhibitor in HPV-positive cervical cancer cell lines (SiHa and HeLa) was found to restore β-catenin accompanied by the downregulation of *FSCN1* expression pattern, thus inhibiting cell invasion ability of these cancer cell lines [122]. In addition, a study in Iran was carried out to investigate the prevalence of HPV and *FSCN1* in cervical squamous cell carcinoma and found an association between *FSCN1* overexpression and HPV positivity [123]. The association of HPV with *FSCN1* was also reported by our group [124], where we demonstrated that E6/E7 oncoproteins of HPV is associated with *FSCN1* overexpression in human colorectal cancer. These studies clearly show that human oncoviruses can deregulate the expression patterns of *FSCN1*, thereby promoting cancer progression (Figure 1). 

## 4. Fascin in Gynecological Cancers 

Numerous studies have reported overexpression of *FSCN1* in various gynecological cancers [123,125,126,127,128,129,130,131,132,133,134,135,136,137,138,139,140,141,142,143,144,145,146,147] as indicated in Table 1.

### 4.1. Fascin in Ovarian Cancer: A Candidate Biomarker and Potential Therapeutic Target

A previous report revealed higher FSCN1 expression in borderline and malignant ovarian tumors as compared to benign cases; however, no significant difference was reported between FSCN1 staining in borderline and malignant cases [147]. In contrast, another investigation reported enhanced expression of FSCN1 in primary, borderline, and metastatic ovarian cancers compared with the normal ovarian tissues where no FSCN1 expression was observed. The authors found that FSCN1 expression was associated with the increased risk of intraperitoneal tumor growth and spread [131]. In addition, the authors detected elevated FSCN1 expression in cell cultures derived from patients with stage IV ovarian cancer compared with cell cultures derived from stage II-III ovarian cancer patients [131]. In another study, Daponte et al. [127] showed FSCN1 expression in advanced poorly differentiated serous ovarian cancer that was associated with poor prognosis, suggesting FSCN1 as an independent prognostic biomarker. More interestingly, an IHC analysis using TMAs to analyze the expression of six EMT biomarkers (FSCN1, cortactin, survivin, EGFR, MMP-2, and MMP-9) in serous carcinomas, mucinous carcinomas, endometrioid adenocarcinomas, and clear cell carcinomas found significant expression of only FSCN1, cortactin, survivin, and EGFR [138]. The study also reported higher scoring for FSCN1 in mucinous carcinomas, which was associated with TNM stage and poorer survival rate [138]. Another study also reported upregulated expression of FSCN1, cortactin, and EGFR in TMAs of four ovarian carcinomas (serous carcinoma, mucinous carcinoma, endometrioid adenocarcinoma, and clear cell carcinoma) [139]. Moreover, FSCN1 overexpression was associated with advanced cancer stage, poorer histological differentiation, and survival rate of mucinous carcinoma, suggesting a potential role of FSCN1 as a candidate biomarker for aggressive serous and mucinous carcinomas [139]. On the other hand, previous studies also reported overexpression of FSCN1 in epithelial ovarian cancer and indicated the interaction between cell and matrix as a vital step in the progression of malignant epithelial ovarian neoplasms [130,134]. Moreover, high FSCN1 scoring was associated with poorer tumor differentiation in serous, mucinous, and endometrioid adenocarcinoma, indicating a role of FSCN1 in analyzing tumor aggressiveness, and was suggested as an independent prognostic risk factor in mucinous carcinoma [138]. Similarly, Coa et al. [126] revealed enhanced expression of FSCN1 in primary mucinous carcinomas in comparison with borderline mucinous tumors with a significant expression in metastatic tumors as compared with primary tumors. Furthermore, while FSCN1 expression was significantly upregulated in borderline and malignant ovarian tumors, there was no expression of FSCN1 in benign ovarian tumors [125]. Another immunohistochemical study revealed that a strong FSCN1 positivity was associated with serous subtype and micropapillary growth pattern [128]. Another investigation by Kostopoulou et al. [137] analyzed the expression of FSCN1 in ovarian cancer using IHC and Western blotting and reported an upregulation of FSCN1 expression in invasive ovarian carcinomas as compared with borderline tumors and cystadenomas. In addition, the study pointed out an association between FSCN1 overexpression and advanced stage and aggressive phenotype [137]. Thus, evaluating FSCN1 expression as a biomarker depicting the progression and outcomes of several types of gynecological cancers has been the center of renewed interest. Another investigation found significantly higher stromal FSCN1 expression in borderline and malignant epithelial ovarian tumors in comparison to normal ovaries and benign epithelial ovarian tumors [132].

McGuire and colleagues showed that the silencing of FSCN1 in ovarian cell lines (HeyA8, Ovcar5, and Tyk-nu), primary human cancer-associated fibroblasts and primary human omental mesothelial cells reduced metastasis [140]. TMA analysis showed higher FSCN1 expression in the tumor stroma than in cancer compartments, and this was associated with the advanced tumor stage [140]. In vitro and in vivo data showed that the loss of FSCN1 significantly inhibited trans-mesothelial migration of the ovarian cancer cell line ES-2 and reduced the interaction between ovarian cancer cells and mesothelial cells in the mouse peritoneal cavity [148]. Moreover, overexpression of FSCN1 in SKOV3 (ovarian cancer cell line) triggered trans-mesothelial migration [148]. On the other hand, in mature and immature neural components, the expression of FSCN1 was detected regardless of rosette formation in immature teratomas derived from both human ovary stem cells, indicating FSCN1 immunostaining as a potential biomarker in diagnosing and grading human immature teratomas [146]. 

A recent study examined the effect of curcumin against FSCN1 in the ovarian cancer cell line SKOV3 and found curcumin to inhibit STAT3 via the JAK/STAT3 signaling pathway. Notably, the inhibition of STAT3 also led to FSCN1 activity inhibition [149]. In addition to blocking FSCN1, in curcumin-exposed ovarian cancer cells, the formation of filopodia was disrupted, and cell migration was reduced [149]. Recently, Yoshihara et al. [148] documented the importance of filopodia in the trans-mesothelial migration of ovarian cancer cells. Additionally, in athymic nude mice, FSCN1 activity was inhibited therapeutically with the compound G2 [140]. The treatment inhibited the actin-bundling into stress fibers as well as ovarian cancer cell migration by reducing GTP-bound Cdc42 and Rac1, further indicating a therapeutic role of G2 in ovarian cancer [140,150].

### 4.2. Fascin in Endometrial Cancer: A Potential Biomarker and Therapeutic Target

Similar to ovarian cancer, studies were performed to detect FSCN1 expression in uterine cancer. Uterine carcinosarcoma cases were assessed for FSCN1 expression using IHC; while FSCN1 was absent in benign cases, it was present in both malignant epithelial and mesenchymal elements of uterine carcinosarcomas. This finding was associated with a more aggressive phenotype (advanced stage and large tumor size) and a poor outcome [142]. Additionally, FSCN1 was found to be a potential IHC biomarker in differentiating uterine leiomyosarcoma from leiomyoma [135]. In undifferentiated endometrial carcinomas, the studies reported a loss of E-cadherin and β-catenin and overexpression of FSCN1, galactin-3, cyclin D1, and p16, which is involved in EMT and invasion, thus contributing to aggressive behavior and poor prognosis [141,143,151]. Another investigation revealed significant overexpression of FSCN1 in proliferative endometrial carcinoma samples as compared with the control samples with a significant association with tumor grade and neural invasion [129]. In another report, Kabukcuoglu et al. [133] showed that during endometrial neoplasia development, there was a loss of stromal FSCN1 expression and its increase in the epithelial compartment; this finding was associated with tumor grade and overall survival. The overexpression of FSCN1 protein was also reported in vulvar cancer; however, immunostaining failed to distinguish in situ from invasive lesions as well as putative HPV-associated and HPV-independent squamous cell carcinomas [145]. 

### 4.3. Fascin in Cervical Cancer: A Potential Biomarker and Therapeutic Target

**Figure 2:**
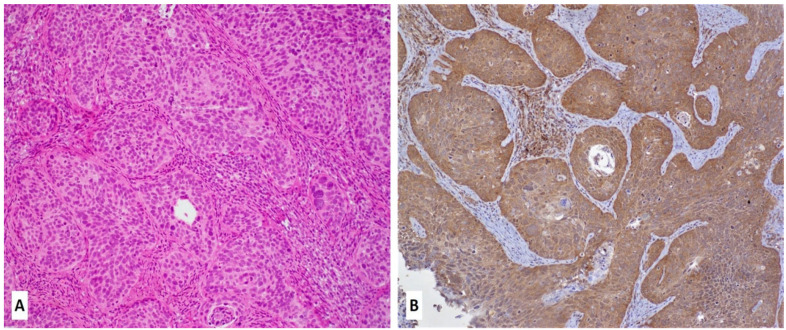
Fascin expression in cervical cancer (**A**,**B**). A case of invasive squamous cell carcinoma of the uterine cervix: (**A**) hematoxylin and eosin slide (20×) with diffused and strong immunohistochemical expression of the fascin protein in cancer cells (**B**, 20×).

In spite of the confirmed role of FSCN1 in several human carcinomas, there has been a limited number of investigations pertaining to the presence and role of FSCN1 in cervical cancers. In this context, Stewart et al. analyzed FSCN1 expression by IHC in in situ and invasive adenocarcinoma of the endocervix and found FSCN1 overexpression to occur during the development and progression of some endocervical neoplasms, indicating the role of FSCN1 in tumor invasion [144]. Koay et al. [136] reported the expression pattern of FSCN1 in cervical carcinoma by IHC; the normal endocervical epithelium was negative for FSCN1, while the normal squamous epithelial stained positive for FSCN1 in basal and parabasal cells [136]. Furthermore, cervical endothelial cells had constant FSCN1 staining, whereas, in CIN lesions and invasive squamous cell carcinomas, there was high FSCN1 expression [136]. Both studies indicate FSCN1-induced invasion in cancer cells due to loss of cell-to-matrix adhesion [132,136]. In addition, in our laboratory, we found FSCN1 to be overexpressed in cervical cancer tissue (Figure 2).

## 5. Fascin in Other Cancers

Several previous studies analyzed *FSCN1* expression using PCR and immunohistochemistry (IHC), revealing its increased expression compared to normal tissues. A systematic review and meta-analysis of studies analyzing the relevance of *FSCN1* in five different carcinomas (breast, colorectal, esophageal, gastric, and lung) by IHC reported that FSCN1 correlates with a high risk of disease progression in breast and colorectal cancers [48]. They also noted that FSCN1 expression is associated with a high risk of mortality in breast, colorectal, and esophageal cancers. Moreover, FSCN1 expression is linked with a high risk of distant and lymph node metastasis in colorectal and gastric carcinomas [48]. 

Of the gastrointestinal cancers, esophageal cancers has the worst prognosis. FSCN1 is involved in the pathogenesis and metastasis of esophageal carcinoma; high FSCN1 expression increases gradually from the normal to the invasive form and correlates with cell proliferation, lymph node invasion, metastasis, and high tumor stage [152,153,154,155,156]. Moreover, overexpression of FSCN1is associated with poor overall and disease-free survival [155]. On the other hand, gastric carcinoma studies using TMA and IHC reported FSCN1 overexpression at both mRNA and protein levels [157,158]; high FSCN1 expression is associated with tumor size, poorly differentiated tumors, invasion, metastasis, TNM stage, and poor survival [157,159,160,161,162,163]. In the colon as well, FSCN1 expression is higher in sporadic and familial colorectal adenomas and adenocarcinomas as compared to the healthy colon [164]; FSCN1 expression was reported to progress from focal during the early stages to diffused in the advanced stages [165]. Increased FSCN1 expression is associated with poor clinicopathological outcomes including advanced tumor stage, grade, and lymph node invasion with poor overall survival and disease-free survival rates [48,166,167]. Therefore, FSCN1 is suggested as a poor prognostic marker for regional and distant metastasis [167,168]. Moreover, FSCN1 expression is also reported in K-ras mutant tumors [169]. FSCN1 expression is also higher in hepatocellular carcinoma tissues compared with normal liver tissues which significantly correlates with the tumor grade, lymph node invasion, and distant metastasis in addition to poor prognosis [170,171,172]. However, a study by Lin et al. showed no correlation between FSCN1 overexpression and clinicopathological features [171]. Similar to all gastrointestinal cancers, in pancreatic cancer, there is an increase in FSCN1 expression during carcinogenesis progression (from pancreatic intraepithelial neoplasia to pancreatic adenocarcinoma); high FSCN1 expression correlates with higher histological grades, and poor overall survival [40,41,173].

While lymphocytes, myeloid, and plasma cells stain negative for FSCN1, in human hematologic malignancies including HIV-related lymphoid hyperplasia, Reed-Sternberg cells, Hodgkin’s lymphoma, Castleman’s disease, and other lymphoid hyperplasia, FSCN1 is overexpressed [174,175]. A study by El Kramani et al. [176] determined FSCN1 levels in with acute myeloid leukemia (AML) and acute lymphoblastic leukemia (ALL) cases in comparison to controls using enzyme-linked immunosorbent assay in the plasma and leukocytes and found FSCN1 expression significantly elevated in AML but not in ALL cases, suggesting FSCN1 as a potential biomarker for AML. Another study analyzed differential expression of FSCN1 in classic Hodgkin lymphoma (CHL), anaplastic large cell lymphoma (ALCL), and diffused large B-cell lymphoma (DLBCL); the study showed that FSCN1 is significantly upregulated in CHL as compared to DLBCL and ALCL, indicating a role of FSCN1 in the differential diagnosis of CHL against ALCL and DLBCL [177].

## 6. Therapeutic Potential of Fascin

Due to the ability of *FSCN1* to induce cell migration, invasion, and metastasis, *FSCN1* is a potential candidate molecule for anti-cancer or anti-metastatic therapy in human cancers [19,36,48,50,73,115,178]. Several targets for *FSCN1* are being developed and include miRNA, siRNA, shRNA, small molecule inhibitors, and nanobodies [73,91,92,93,94,95,96,97,99,140,150,179,180,181,182,183,184,185,186,187,188,189].

In multiple types of cancers, in vitro studies demonstrated that overexpression of miRNAs targeting *FSCN1* in cancer cell lines reduce cell growth, proliferation, migration, and invasion [91,92,93,94,95,97,99], thus indicating miRNAs or miRNA-based reagents as potential therapeutic options. On the other hand, siRNAs specifically degrade targeted mRNA; in vitro and in vivo studies reported *FSCN1* knockdown by siRNA leading to decreased cell migration and metastasis, respectively [54,73,140,150,182,183]. Over the last 10 years, more than 50 therapeutic RNAi-based drugs entered phase- I, II, and III trials, of which 15 phase- I, II, and III programs are dedicated to cancer treatment [190]. While miRNAs and siRNAs can be developed as therapeutics for metastatic cancers, there are several limitations in their role in clinical settings. Overcoming the key challenges, including adverse side-effects, delivery systems and administration paths, dosage concerns, and off-target effects, is necessary to develop RNAi-based therapies for cancer and other diseases [191]. Multiple immune-related side-effects and severe hyperbilirubinemia were some of the adverse events that developed during clinical trials of miRNA-based therapies [192,193,194]. Even though several preclinical studies use mouse models of cancer, only few miRNA candidates have reached clinical phases. Further investigations, including pharmokinetic studies in animal models, are essential to understand the role of RNAi-based therapies in humans [191]. On the other hand, nanotechnology aims to provide multipurpose platforms to allow safe biomolecule delivery, enhance therapeutic efficacy, reduce drug dosage, and minimize adverse events [195]. However, these systems are yet to reach human trial phase as nanocarrier application is dependent on various parameters (average diameter, charge, shape, surface chemistry, and polydispersity index) [195]. In solid tumors, although nanoparticles are stabilized, their mechanistic entry is more complex plausibly due to the involvement of trans-endothelial pathways [196,197]. Moreover, establishing the optimal dose in RNAi-based therapy is complex as treating patients with either a non-active or potential toxic dose is unethical [191]. Since initial doses for phase I/II trials are derived from in vitro and in vivo data (preclinical), various variables including size, volume, immune response, administration routes, and toxicity are major areas of concern [191,198]. It is worth noting that, while RNAi-based therapy is frequently administered intravenously or subcutaneously, development of oral therapy is essential for clinical trials [199]. Finally, RNAi-based therapeutics come with a high cost to cover both RNAi-based products and emerging nanocarriers as compared to prevailing anti-cancer therapeutics; therefore, the cost–benefit ratio is another challenge involved in this kind of therapy [200]. 

Experiments using inhibitory nanobodies against FSCN1 protein showed disruption of the FSCN1/actin-bundling [187]. Although in vitro studies using FSCN1-specific nanobodies in breast (MDA-MB-231) and prostate (PC3) cancer cells inhibited the formation of invadopodium and cell invasion [187], the use of FSCN1-specific antibodies in clinical settings needs to be established. Moreover, series of thiazole derivatives, isoquinolone, and pyrazolo[4,3-c] pyridine were also reported to be potential inhibitors of metastasis by targeting FSCN1 [181,189].

Likewise, small molecule inhibitors can reduce tumor cell migration and invasion and help pave the way against *FSCN1*-induced tumors [75,179,184,185,186]. In ovarian cancer, Wang et al. treated the ovarian cancer cell line (ES-2) with a Leucine aminopeptidase 3 (LAP3) inhibitor, bestatin [188]. Bestatin was found to significantly inhibit tumor cell migration and invasion by blocking *FSCN1* promoter and reducing its expression, thus acting as a plausible anti-metastatic therapeutic agent [188]. Other studies have shown that migrastatin and its analogues target *FSCN1* and block its activity, thereby reducing cell migration, invasion, and tumor metastasis [179,185]. In addition to migrastatin, another investigation in colorectal cancer cells showed an antimigratory and anti-invasive effect of imipramine (anti-depressant) by inhibiting *FSCN1* activity [75], thus introducing a novel molecular targeted treatment in *FSCN1*-induced tumors. A novel small molecule compound G2 and its derived analogs (NP-G2-011, NP-G2-036, NP-G2-044, and NP-G2-050) were tested and displayed anti-metastatic properties along with enhanced response and survival in in vivo models by blocking *FSCN1* activity [184,186]. Recently, the phase 1A clinical trial in ovarian cancer patients was carried out to evaluate the dosage and safety of NP-G2-044; the drug was administered daily as a single oral dose (200–2100 mg) for 6 weeks, including four weeks of daily dosing and two weeks rest period [180]. While no dose-limiting toxicity and fatality were reported, the trial demonstrated the inhibitor (NP-G2-044) as a safe single-drug, with a daily dose of 1600 mg as the provisional recommended phase 2 dose [180]. Prior to treatment with the drug (NP-G2-044), ovarian cancer patients with metastasis to visceral organs were treated with anti-cancer therapeutic drugs. Treatment with NP-G2-044 showed a comparitavely better treatment efficacy as compared to treatment with anti-cancer therapeutic modalities [180]. Moreover, the drug displayed anti-tumor and anti-metastatic properties including progression-free-survival and metastasis-free interval, particularly for metastatic ovarian cancer patients [180]. Proposed future studies include a phase 2A clinical trial to assess the efficacy of NP-G2-044 at the identified dose (1600 mg), both in monotherapy as well as in combination with anti-PD-(L)1 immune checkpoint inhibitors [180]. Table 2 summarizes anti-fascin-based therapeutic approaches. 

Since *FSCN1* can stimulate cancer cell migration and metastasis, there are several limitations in developing therapeutic targets against *FSCN1*. While *FSCN1* is not expressed in adult epithelial tissues, it is normally expressed in other adult non-epithelial tissues [14], raising the concern that *FSCN1* inhibitors may have negative side effects. Specifically, the inhibition of *FSCN1* may cause neuronal, kidney, endocrine, wound healing, and immune defects.

## 7. Conclusions

*FSCN1* is regulated by several signaling pathways (AMPK/mTOR, Wnt/β-catenin, and MAPK) and is overexpressed in various human carcinomas including gynecological cancers; however, understanding the exact molecular mechanisms underlying *FSCN1* deregulation and interaction with other genes and oncoviruses, especially in gynecological cancers, is still nascent. Although several studies have indicated a potential diagnostic utility of *FSCN1*, its therapeutic role as an anti-cancer target is still under investigation. We believe that further studies are needed, including the development of conditional transgenic and/or knockout animal models, to determine the role of *FSCN1* targeting as a potential therapeutic route for gynecological carcinomas.

## Figures and Tables

**Table 1:** Fascin Overexpression in Gynecological Cancers and its Association with Clinico-pathological Features.

Type of Cancer	Detection Method (Assay)	Clinicopathological Features	References
Ovarian Cancer	IHC	Poor overall survival and prognosis	[127]
	IHC	Serous subtype, micropapillary pattern, FIGO stage, and risk of recurrence	[128]
	IHC	Serous subtype and residual postoperative tumor	[130]
	ICC and IHC	Involved with intraperitoneal invasion	[131]
	IHC	Presence of vascular invasion, psammomatous calcifications, and lymphocytic infiltration	[132]
	IHC	-	[134]
	IHC and WB	Tumor aggressiveness	[137]
	IHC	-	[125]
	IHC	-	[126]
	IHC	T and Nstage, AJCC clinical stage, and poor survival rate	[138]
	IHC	Advanced TNM stage, poor histological differentiation, and poor survival rate	[139]
	IHC and IF	Invasion and migration, metastasis, colonization, and poor prognosis	[140]
	IHC	Tumor grade and tumor aggressiveness	[147]
Endometrial Cancer	IHC	Tumor grade and neural invasion	[129]
	IHC	High tumor grade	[133]
	IHC	Tumor aggressiveness, distant metastasis, and local recurrence	[141]
	IHC	Lymphovascular space invasion and epithelial-mesenchymal transition	[143]
	IHC	Higher expression in leiomyosarcoma	[135]
	IHC	Extrapelvic disease, higher stage, larger tumor size, shorter progression-free interval, and reduced ER-α expression	[142]
Vulvar Cancer	IHC	-	[145]
Cervical Cancer	IHC	Increased invasivion	[136]
	IHC	Tumor invasion	[144]
	NM-PCR and IHC	HPV overexpression	[123]

ICC: immunocytochemistry; IF: immunofluorescence; IHC: immunohistochemistry; NM-PCR: nested multiplex polymerase chain reaction; WB: Western blot.

**Table 2:** Anti-fascin-based Therapeutic Approaches.

Therapeutic Approach	Outcome	Reference
FASNb5 (Fascin nanobody, Kd~35 nM, 1:1 stoichiometry)	Invadopodium instability	[187]
CORNb2 (Cortactin nanobody, Kd~75 nM, 1:1 stoichiometry)	Blocks invadopodium precursor formation and MMP secretion	[187]
Migrastatin and its analogues	Inhibits cell migration, invasion, and metastasis	[179,185]
Thiazole derivatives	Inhibits cell migration and suppresses angiogenesis	[189]
Bestatin (LAP3 inhibitor)	Inhibits FSCN1 expression and suppresses tumor cell migration and invasion in a dose-dependent manner	[188]
Isoquinolone and pyrazolo[4,3-c]pyridine inhibitors	Disrupts actin binding	[181]
G2 compound	Inhibits actin structures, migration, and invasion of cancer cells	[186]
NP-G2-044	Increase in duration of treatment, progression-free-survival, and metastasis-free interval. Displays anti-tumor and anti-metastatic activity	[180,184]
NP-G2-044 and PD-L1 inhibitor	In progress	[180]
Curcumin	Blocks fascin expression through JAK/STAT3 pathway downregulation. Inhibits cell attachment, invasion, and migration	[149]

## Abbreviations

Abl: Tyrosine-protein kinase ABL1; ABS: actin-binding site; AhR: aryl hydrocarbon receptor; ALCL: Anaplastic large cell lymphoma; ALL: Acute lymphoblastic leukemia; AML: Acute myeloid leukemia; AMPK: 5’ adenosine monophosphate-activated protein kinase; Cdc: Cell division control protein 42 homolog; CHL: Classical Hodgkin lymphoma; CREB: cAMP-response element binding protein; DLBCL: Diffuse large B-cell lymphoma; EBV: Epstein-Barr virus; EGF: Epidermal growth factor; EGFR: Epidermal growth factor receptor EMT: Epithelial-mesenchymal transition; ERK: Extracellular regulated kinase; FSCN: Fascin; GSK-3β: Glycogen Synthase Kinase-3 Beta; GTP: Guanosine triphosphate; HIF-1α: Hypoxia-inducible factor-1-alpha; HPV: Human papillomavirus; HTLV-1: human T-lymphotropic virus type-1; IHC: Immunohistochemistry; IL: Interleukin; JNK: c-Jun N-terminal kinase; kDa: Kilo Dalton; LAP3: Leucine aminopeptidase 3; LMP: Latent membrane protein; MAPK: Mitogen-activated protein kinase; miRNA: microRNA; MMP: Matrix metalloproteinase; mTOR: Mammalian target of rapamycin; NF-κB: Nuclear factor kappa light chain enhancer of activated B cells; NT2: NTERA-2; Rac1: Ras-related C3 botulinum toxin substrate 1; SCID: Severe combined immunodeficiency; Ser: Serine; shRNA: Small hairpin RNA; siRNA: Small interfering RNA; SMAD4: SMAD family member 4, Mothers against decapentaplegic homolog 4; SNAI2: Snail Family Transcriptional Repressor 2; SNAIL: Zinc finger protein SNAI1; Sp1: Specificity protein 1; Src: Proto-oncogene tyrosine-protein kinase sarcoma; STAT3: Signal transducer and activator of transcription 3; TCF: T-cell factor; TEAD: Transcriptional enhanced associate domain; TFs: Transcription factors; TMA: Tissue microarray; TNF: Tumor necrosis factor; UTR: Untranslated region; Wnt: Wingless; YAP: Yes-associated protein.
